# Injurious Effect of Tea on Nursing Children

**Published:** 1899-10

**Authors:** 


INJURIOUS EFFECT OF TEA ON NURSING CHILDREN.—It sometimes occurs that what is supposed to be scurvy in the mouth of a nursing child, can be attributed to the excessive tea drinking of its mother. The child will have every appearance of the spongy gums, bleeding, with fetor of the breath, purple spots over the posterior aspect of one or both arms and legs, and some of these spots may be as large as the palm of the hand, with yellow borders as though the child had been beaten or had been subjected to a fall or otherwise bruised. Beneath the spots the tissues present an indurated surface. In such cases the mother should be questioned as to her diet, and it is likely you will find that she uses strong tea in excess. You will often be astonished to hear that she is an inveterate tea drinker. Not alone does she drink tea at meal time, but between meals, and in all probability, a bowl at bedtime.

In such cases a change of diet, together with a suitable mouthwash, is all that the case requires.

